# Climate Change Influences on the Global Potential Distribution of Bluetongue Virus

**DOI:** 10.1371/journal.pone.0150489

**Published:** 2016-03-09

**Authors:** Abdallah M. Samy, A. Townsend Peterson

**Affiliations:** 1 Biodiversity Institute, University of Kansas, Lawrence, Kansas, 66045, United States of America; 2 Entomology Department, Faculty of Science, Ain Shams University, Abbassia, Cairo, Egypt; Institut National de la Recherche Agronomique (INRA), FRANCE

## Abstract

The geographic distribution of arboviruses has received considerable attention after several dramatic emergence events around the world. Bluetongue virus (BTV) is classified among category “A” diseases notifiable to the World Organization of Animal Health (OIE), and is transmitted among ruminants by biting midges of the genus *Culicoides*. Here, we developed a comprehensive occurrence data set to map the current distribution, estimate the ecological niche, and explore the future potential distribution of BTV globally using ecological niche modeling and based on diverse future climate scenarios from general circulation models (GCMs) for four representative concentration pathways (RCPs). The broad ecological niche and potential geographic distribution of BTV under present-day conditions reflected the disease’s current distribution across the world in tropical, subtropical, and temperate regions. All model predictions were significantly better than random expectations. As a further evaluation of model robustness, we compared our model predictions to 331 independent records from most recent outbreaks from the Food and Agriculture Organization Emergency Prevention System for Transboundary Animal and Plant Pests and Diseases Information System (EMPRES-i); all were successfully anticipated by the BTV model. Finally, we tested ecological niche similarity among possible vectors and BTV, and could not reject hypotheses of niche similarity. Under future-climate conditions, the potential distribution of BTV was predicted to broaden, especially in central Africa, United States, and western Russia.

## Introduction

The global distribution of arboviruses has received considerable attention from public health organizations after recent emergence events in several parts of the world [1,2]. Bluetongue virus (BTV) is an arboviral disease in ruminants [3], caused by a member of the genus *Orivirus* in the family *Reoviridae*. The disease is transmitted among ruminants by the bites of biting midges of the genus *Culicoides* [4].

BTV has been responsible for massive sheep mortality; for example, outbreaks in the Mediterranean region since 1998 resulted in deaths of over 800,000 sheep [5]. A single strain of BTV in Belgium disrupted animal trade and killed animals with a market value of UK £180 million during a 2006–2007 outbreak [6]. In the United States, BTV causes losses of US $125 million yearly [7]. Previous reports have discussed early introduction of infected sheep into South Africa 125 years ago [8], but others identified South Africa as the origin of the infection [9].

BTV geography was long limited to a range between 40°N and 35°S [1]. Recently, however, several BTV strains began to spread worldwide [1,10,11], including to more northern parts of Europe, in 1998 [4]. The expansion and the potential for susceptibility of new vector species to the virus raises concerns of broader BTV spread [4,12–14]. BTV is transmitted by several vector species: *Culicoides imicola* Kieffer, 1913 (Diptera: Ceratopogonidae) is the most significant vector in the Old World [15], but three other species serve as vectors in the United Sates alone [16]. The distribution and movement of hosts has also been identified as a limiting factor for BTV spread; although BTV is known to have infected several ruminants, cattle and sheep are identified as primary reservoirs in several endemic areas worldwide [1]. The combination of climate, presence of susceptible host, and presence of competent vectors marks areas where BTV can circulate in the long term among livestock.

Previous studies have mapped BTV risk based on occurrence data from single countries [17–19]; others included vector distributions in mapping efforts [5,18]. One study explored the global distribution and possible future shifts in the distribution of *C*. *imicola* across the world [15]. Ecological niche models provide a robust approach by which to assess and evaluate distribution of disease risk [20]: this approach has been used in mapping everything from fungal to arboviral diseases in several recent analyses [21,22].

Here, we developed a comprehensive database of BTV case occurrences, and estimated the global potential distribution of BTV under both current and future climate conditions. The study used outputs from 62 general climate models (GCMs) and four representative concentration pathway (RCP) scenarios from the Fifth Assessment Report (AR5) of the Intergovernmental Panel on Climate Change (IPCC) to estimate the future potential distribution of the virus. Finally, we tested niche similarity between several vector species and BTV case distribution in different geographic areas to provide some level of assessment of the role of particular potential vector species in BTV transmission.

## Materials and Methods

### Input data

Primary records of BTV occurrences (i.e. data reports of animal infections) were obtained from the PubMed database and Web of Knowledge using the search term “bluetongue virus”, as well as from OIE reports (www.oie.int), the ReoID database (www.reoviridae.org/), and the Food and Agriculture Organization Emergency Prevention System for Transboundary Animal and Plant Pests and Diseases Information System (EMPRES-i; http://empres-i.fao.org). Data regarding BTV occurrences through November 2014 were used in calibration, whereas occurrences from after that date (through September 2015) were used to provide a semi-independent data set for model evaluation (see Discussion). BTV records were drawn from diverse sources as we are seeking a global map of disease across the world; however, OIE and FAO data are limited to countries where BTV is notifiable, with most sampling in Europe and United States; data did not include the older BTV outbreaks in Africa and Asia. For niche comparisons with possible vectors, we collected vector occurrences from the Global Biodiversity Information Facility (GBIF; www.gbif.org) and literature in the PubMed and Web of Knowledge databases. The vector occurrences included records for six species: *C*. *imicola*, *C*. *insignis* Lutz, 1913, *C*. *variipennis* Coquillett, 1901, *C*. *sonorensis* Wirth & Jones, 1957, *C*. *occidentalis* Wirth & Jones, 1957, and *C*. *brevitarsis* Kieffer, 1917. When geographic references were textual, we assigned geographic coordinates based on consultation of online gazetteer data (www.gpsvisualizer.com). Data were filtered to eliminate duplicate records; we further reduced the data such that no pair of points was separated by <20 km (i.e., a single pixel) to reduce biases in calibrating ENMs [23]. The final occurrence data set was divided in two equal portions: half to calibrate the model, and half for evaluating model predictions.

To characterize current global climates, we used data available from the WorldClim archive (www.worldclim.org), which comprise19 bioclimatic variables derived from monthly temperature and rainfall values collected during 1950–2000 [24]. We used the 10ˈ spatial resolution in light of the global extent of our modeling efforts. For future conditions, we obtained data based on GCM outputs for 2050. These data comprised four RCPs spanning broadly different emissions scenarios into the future. Our future-climate projections thus summarized 62 combinations (S1 File). We used bioclimatic variables derived from monthly temperature and precipitation values because they are known factors in BTV transmission risk [1,25,26].

We omitted bioclimatic variables 8–9 and 18–19 from analysis, in light of known spatial artifacts in those four variables. The remaining of 15 variables were subjected to a principal components analysis (PCAs) to reduce the dimensionality of our models and avoid multicollinearity of variables (see summary of variable correlations in S2 File). The component loadings in the present-day data were used to transform future-climate data, using the ENMGadgets package [27] in R version 3.2.0 [28].

### Ecological niche modeling

The maximum entropy algorithm implemented in Maxent version 3.3 [29] was used to estimate the ecological niche of BTV, roughly defined as the set of environmental conditions under which the species can maintain populations [20]. Our model was based on the first 6 principal components described above. We estimated the accessible area (**M**) [30,31] considering the geographic distribution of recent BTV outbreaks, which have been very broad, covering much of the world. We used the bootstrap functionality in Maxent to produce 100 replicate analyses. We used the median values across all models and replicates as a best estimate of the ecological niche of BTV. Finally, we calculated the median of the medians across all GCMs within each RCP scenario. Final models were thresholded based on a minimum allowable omission error rate of 5% (*E* = 5%; [32]), assuming that a minimum of 5% of occurrences data may have errors in geolocation that misrepresented environmental values. We used the range (maximum–minimum) as an index of uncertainty between diverse models within each RCP.

### Model robustness

Model robustness was evaluated using partial ROC statistics [20,32], which avoid many of the problems with traditional ROC approach [33]. We used the partialROC function in the ENMGadgets package in R [27] and the 50% subset of available occurrence data described above. A further evaluation of our model was based on independent data from recent outbreaks reported to FAO EMPRES-i. These data represent outbreaks reported between December 2014 and September 2015; that is, the evaluation data come from the year following the temporal span of the data used for model calibration. We used a one-tailed cumulative binomial probability distribution that assessed the probability of obtaining the observed level of correct prediction by a chance alone, given the background expectation of correct predictions based on the proportional coverage of the region by the thresholded model prediction.

### Niche overlap of bluetongue virus and its vectors

We tested the niche similarity between each potential vector species and BTV using the background similarity test implemented in ENMTools version 1.4.4 [34]. We developed a specific **M** hypothesis [31] for each vector species as follows: *C*. *imicola*, a vector of BTV in the Old World [1,5,35], so we estimated a broad accessible area (**M**) that included all of Europe, Asia, and Africa for that species. *Culicoides insignis* is reported from North, Central, and South America [36], so its **M** was estimated to include all of the Americas. The **M** hypotheses for *C*. *variipennis* and *C*. *sonorensis* were estimated as all of North and Central America. *Culicoides brevitarsis* was restricted to East Asia and Australia, and *C*. *occidentalis* was limited to the southern United States and Central America.

The background similarity test assessed whether vector and BTV niches are less similar than expected given the “background” similarity manifested across the accessible areas of each [34]. We compared niche model similarity values based on actual occurrences of each species, with distributions of background similarity based on comparison of the niche of one species with “niche” models based on random points from across the **M** of the other species. We used numbers of random points equal to the number of actual occurrences for the other species. The null hypothesis of niche similarity was rejected if the observed *D* or *I* values for the BTV and vector species in question fell below the 5^th^ percentile in the random-replicate distribution.

## Results

**Fig 1:**
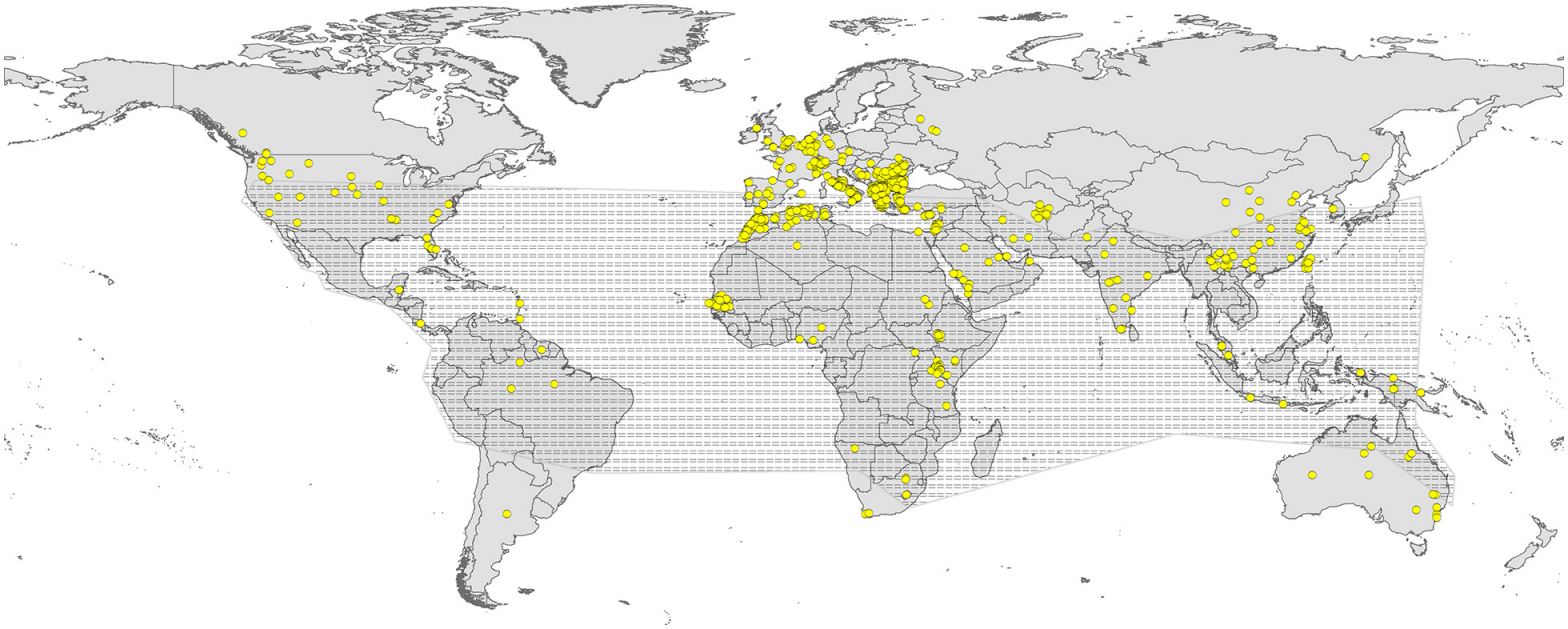
Summary of bluetongue virus occurrences (yellow points) available for model calibration worldwide. Dotted black shading represents the early belt of BTV occurrence.

We assembled a total of 1677 unique occurrences for BTV around the world for model calibration. These points were filtered down to 1260 records in individual pixels. The overall pattern of occurrences indicated a geographically broad distribution of BTV, with more intense sampling efforts in Europe, where the virus invaded recently (Fig 1). Sampling was much more sparse in Africa and South America. Most BTV records were outside the early geographic belt identified for BTV distribution [37] (Fig 1). We also assembled an overall total of 798 occurrence records for six vector species: *C*. *imicola* (*N* = 408), *C*. *sonorensis* (*N* = 239), *C*. *variipennis* (*N* = 75), *C*. *insignis* (*N* = 33), *C*. *brevitarsis* (*N* = 23), and *C*. *occidentalis* (*N* = 20). These species have different ranges across the world (S3 File): *C*. *imicola* has a broader distribution extending from East Asia to western Africa; however, other species are limited in their ranges to East Asia and Australia (*C*. *brevitarsis*), North and Central America (*C*. *variipennis* and *C*. *sonorensis*), southern United States and Central America (*C*. *occidentalis*), and North and South America (*C*. *insignis*).

**Fig 2:**
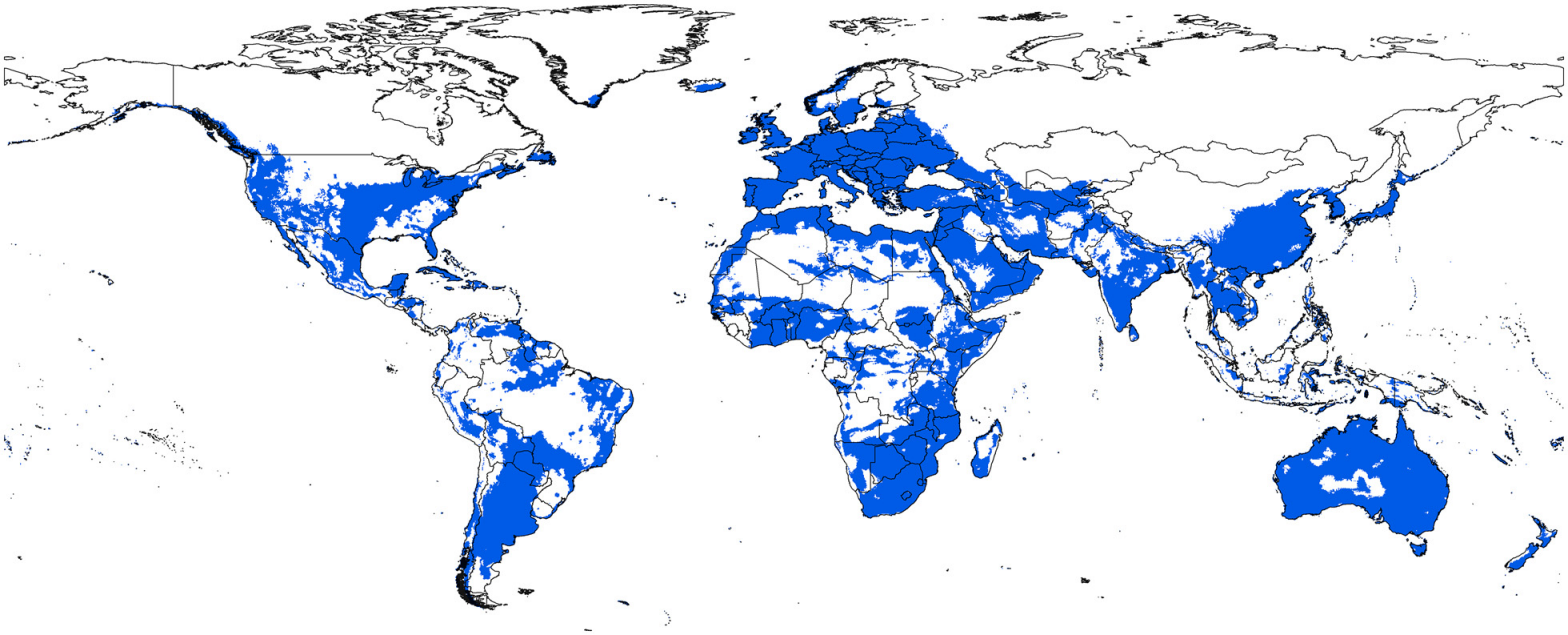
Current potential distribution map for bluetongue virus based on present-day climatic conditions. Blue shaded areas are modeled suitable conditions, and white areas are unsuitable conditions.

**Fig 3:**
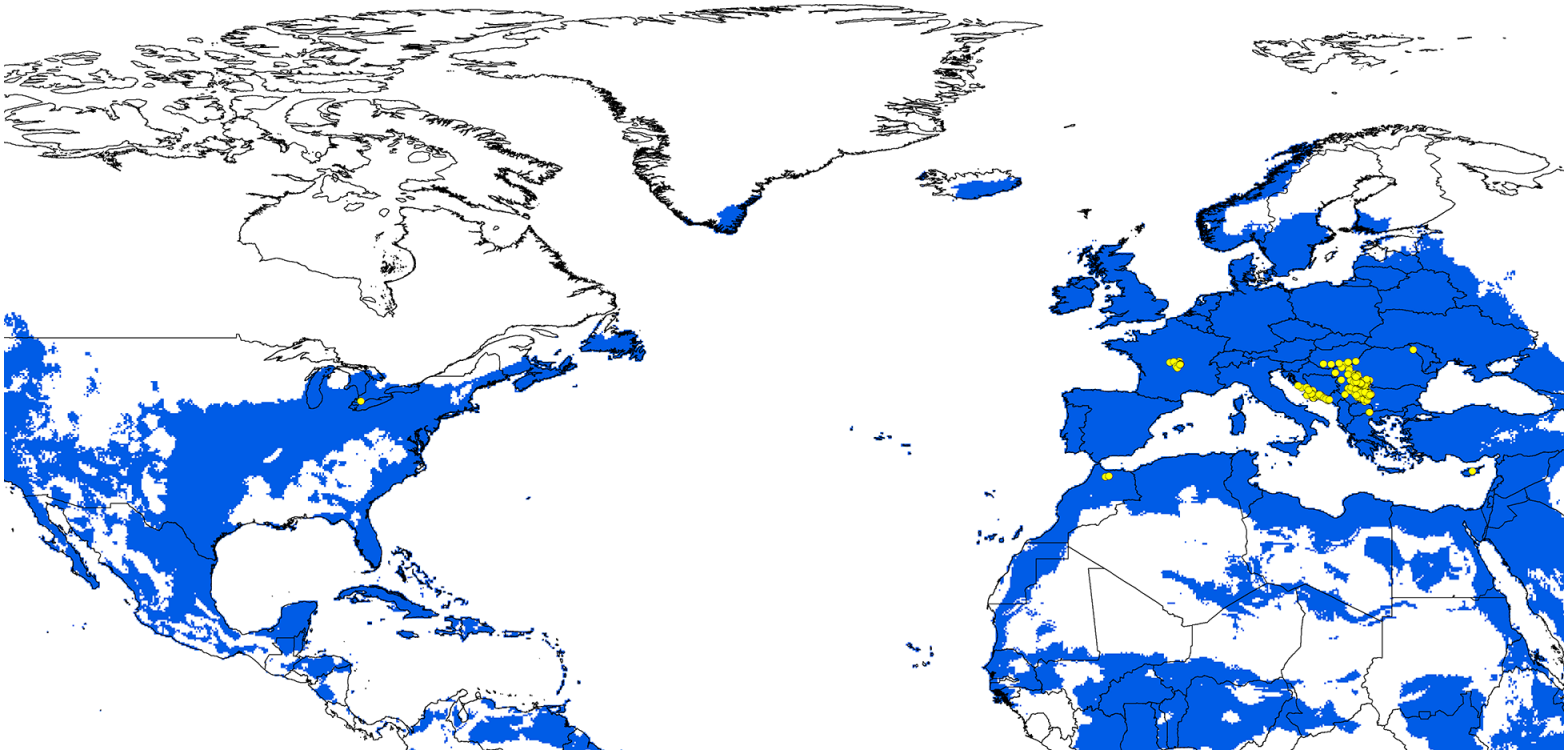
Relationship of additional independent BTV records to areas predicted as suitable for bluetongue virus occurrences. Yellow points are independent BTV occurrence data from the Old World and North America. Blue areas are represented as suitable and white as unsuitable.

The potential distribution of BTV under present-day conditions showed high suitability across southern Europe, Australia, the Indian Subcontinent, and northern and southern Africa (Fig 2). BTV occurred in tropical, subtropical, and temperate climate zones. Suitable areas were also identified in West Africa, United States, and southern and western Canada. In all, the model outputs corresponded well to known areas of transmission around the world. Model predictions were significantly better than random expectations, in that partial ROC AUC ratios were uniformly higher than the random classifier with an AUC ratio of 1 (*P* < 0.01). The data set of 331 independent records was used to evaluate the robustness of our models in anticipating the current outbreaks across southern Europe, North Africa, and the United States. The model was significantly able to anticipate all 331 points reported for the most recent outbreaks of BTV (Fig 3; cumulative binomial test, *P* < 0.0001).

**Fig 4:**
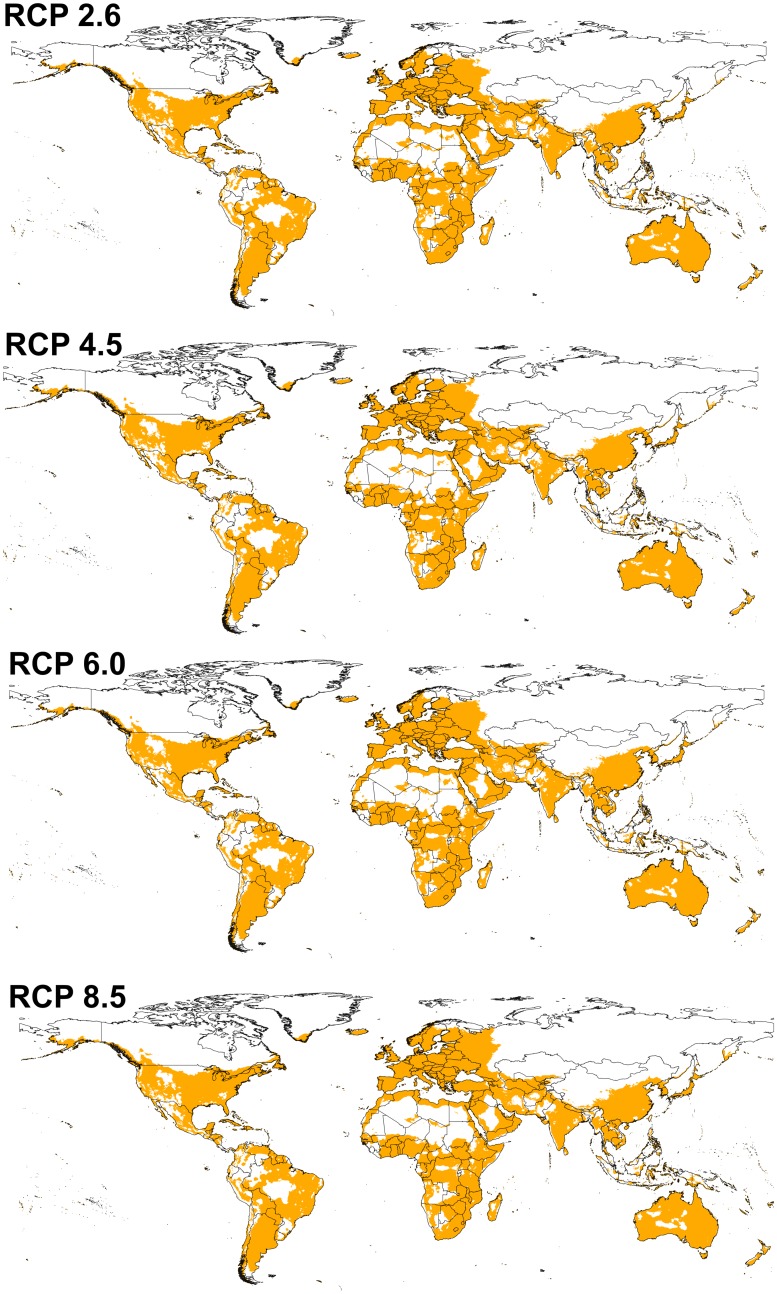
Predicted potential distribution maps for bluetongue virus under future climatic conditions. Models were calibrated across present-day conditions, and transferred to the future climate conditions. Each model is the median of all climate models across each representative concentration pathways (RCPs). Orange areas are modeled suitable conditions; white areas are unsuitable conditions for BTV occurrences.

**Fig 5:**
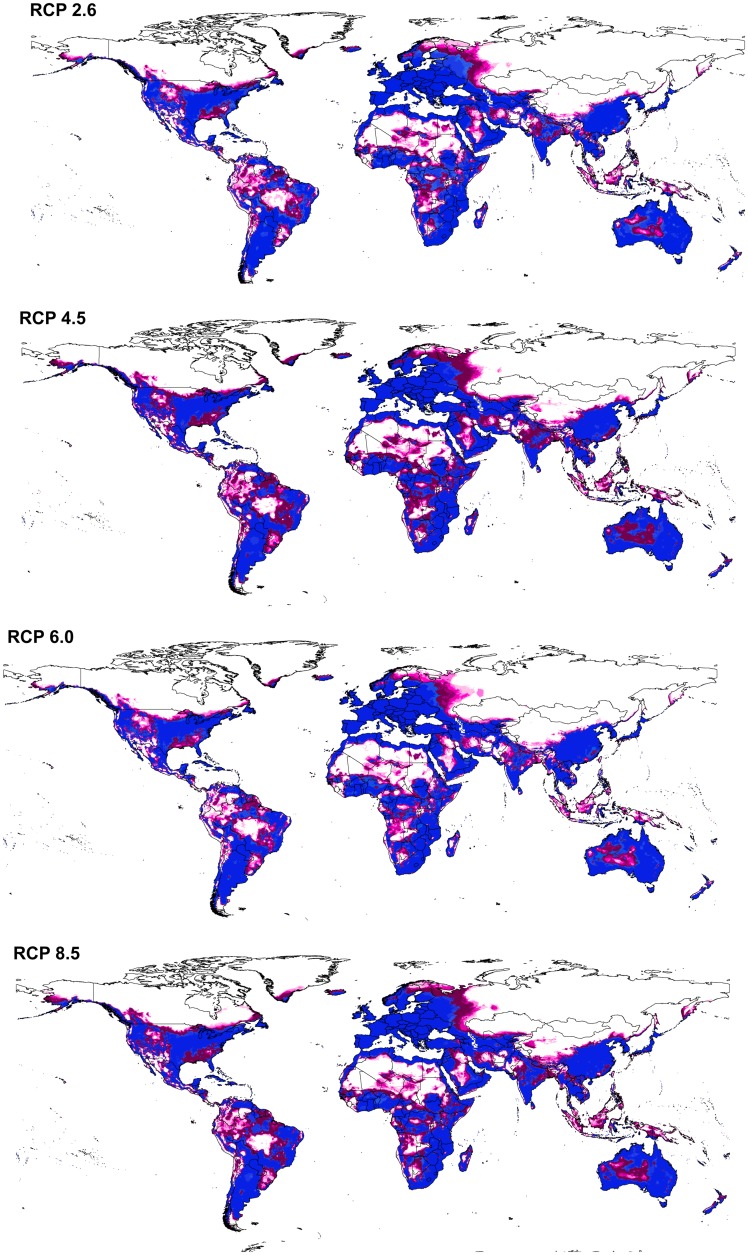
Summary of the modeled global distribution of bluetongue virus under both current and future climatic conditions to show the stability of predictions at present and into the future, and to illustrate differences among representative concentration pathways (RCPs). Dark blue represents model stability under both current and future conditions, light blue represents low agreement between current and future conditions, dark purple represents agreement among all climate models in anticipating potential distributional areas in the future, and light purple indicates low agreement between diverse climate models as regards distributional potential in the future.

BTV model transfers to future conditions indicated a pattern that was overall similar to that estimated for present-day conditions. However, the potential distribution of the virus under future conditions was broader, and included areas not identified as suitable under current conditions (Fig 4). This potential for expansion was particularly notable in central Africa, the United States, and western Russia. Under all future climate scenarios, the virus was seen to have a broader potential geographic distribution than at present (Fig 4). We noted few differences between GCMs within each climate scenario, such that model predictions were consistent over much of the world, with exceptions in western Russia, northern Europe, western South America, and Indonesia, where future projections were less stable (Fig 5).

BTV range increased from RCP 2.6 to RCP 8.5 (potential distributional area increased by 8.11% between present-day and RCP 2.6, and by 9.08% between present-day and RCP 8.5). Differences were also noted in the future potential BTV ranges of different models within each climate scenario (S4 File); the potential distributions under the different model conditions are summarized in the electronic supplementary materials as a GeoTIFF dataset (https://figshare.com/s/ac5383809b411c0f8779).

Finally, we tested the similarity of estimated niches between BTV and vector species, taking into account the background similarity between the accessible areas of each [34]. We could not reject the null hypothesis of niche similarity between BTV and any of the vector species (*P* > 0.05) (S5 File). Hence, present-day environmental conditions occupied by BTV and its vectors were not demonstrably different, and we found no indication of unlinked transmission and vector occurrence.

## Discussion

This study built a comprehensive database of BTV occurrences from 1964 through November 2014. We used these data to map the potential distribution of BTV in the present-day, and also to identify future potential distributional shifts in view of the most recent future climate scenario model outputs. The availability of BTV data was poor until large-scale outbreaks across Europe began in the late 1990s; since then, the disease has caused serious impacts on international animal trade, and serious illness and high mortality rates among ruminants [38,39], and reporting has been more detailed.

Climate has been suggested as a major driver of the distribution of BTV [1]; for example, the European outbreaks were thought to be a consequence of warming climates [1], and the virus expanded another ~800 north in 2005 [40,41]. Historically, the disease was found in a belt between 40°N and 35°S that included northern Australia, parts of the Indian Subcontinent, Middle East, Africa, Cyprus, and the Americas. Prior to that point, BTV was known only from South Africa and Cyprus [8, 42], The origin of the disease thus has three possible explanations: (1) BTV was present in both Africa and Europe but was not documented owing to misidentification or poor diagnostic tools; (2) BTV originated in South Africa and dispersed to Europe; (3) BTV originated in Europe and dispersed to South Africa. Phylogenetic analyses suggest that strains responsible for the new BTV outbreaks are similar to those that circulated for decades in early epidemic sites [1]. FAOSTAT data indicate results revealed continuous international trade between Europe and Africa [43], which could allow viral infections in animals to move between the two continents. This observation also suggests that the virus will be able to spread to new sites that become suitable as climatic conditions change in coming decades.

We modelled BTV occurrences using the most recent version of 62 future climate model outputs, and used four RCPs to summarize variations among possible greenhouse gas concentration trajectories. Our results indicated the possibility of range expansion to other regions where the virus is not presently endemic, in response to climate changes [1].

Previous studies predicted the distribution of BTV and its vectors; most of these studies were limited to Europe after BTV emergence in Europe [5,18,26], and assessed transmission risk based mostly on vector distribution and abundance [5,18]. However, a single study took the advantage of a mechanistic models to quantify *R*_*0*_ values of BTV across Europe based on different temporal scales of climate data. This latter study demonstrated BTV risk areas across most of Europe that coincide with our results. However, our prediction covers a much broader portion of Europe, extending across much of the continent, east to western Russia. A recent study of BTV potential in the United States predicted that further northwards expansion of *C*. *sonorensis* can be expected in the future [44]. Our current study anticipated both the northern United States, southwestern Canada, and Ontario as at risk, as long as both BTV and vector expand distributionally in tandem; however, it marked unsuitability of conditions to virus spread along the US Gulf Coast and in the eastern Rocky Mountains, where livestock and wild animals have been diagnosed as positive to BTV [10]. This discord may reflect limitations of our model; however, these same regions were identified as suitable in our future models, which may indicate that our present-day models cannot anticipate risk in these regions given ongoing climate reorganization.

The models estimated in our study showed significant performance in two tests based on testing data that are ostensibly independent from the data used in model calibration. However, we see some room for concern about this level of independence because disease events are dependent events on a number of levels—individual cases may be linked to one another via pathogen and vector population biology, on both micro and macro scales. Similarly, surveillance is often responsive, and gets concentrated in affected areas, which can create additional dependencies. For these reasons, our data set was selected to constitute different data sources that represent sampling across the world and not just notified data from EMPRES-i. Our evaluation tested the possibility of these models to predict the recent outbreaks across the world from December 2014 until the most recent outbreak in Ontario, Canada. These models could anticipate all of these current outbreaks.

Different levels of uncertainty were associated with the mapping process (S6 File). These uncertainty estimates were based on variations in predicted distributions of BTV across different climate models rather than just an estimate of internal uncertainty for predictions under the same climate model [22,45].

Finally, our models offered an interesting perspective on vector associations of BTV infections around the world. The BTV transmission cycle includes hosting by ruminants and vectors that transmit virus between hosts. Major knowledge gaps include the broader host distribution of BTV in diverse livestock hosts, and the vanishingly few studies that have focused on vector competence of different species of *Culicoides*. This study characterized diversity in the species of vectors associated with BTV in different parts of the world: *C*. *imicola* in the Old World, *C*. *sonorensis* and *C*. *variipennis* in North America, *C*. *insignis* in North and South America, *C*. *occidentalis* in southern United States and Central America, and *C*. *brevitarsis* in Australia. We tested niche similarity between each vector and BTV distribution across the accessible region for the corresponding vector; we could not reject the null hypothesis in any case, so vector populations and BTV appear to share similar ecological niches. This observation is important because (1) vector populations can assist in identifying the potential distribution of the BTV in countries where the disease is not reported dependably to authorities, and (2) vector populations may drive BTV response to climate change [46].

The current study leaves important questions unanswered regarding the global distribution of BTV: one related to the relationship between vector population dynamics and changes in BTV transmission, host response to climate change, and responses of different BTV serotypes to climate and how much these responses are similar or different. Our future work will focus on exploring these questions to illuminate additional key details of BTV epidemiology and ecology around the world.

## Supporting Information

S1 File A summary of four representative concentration pathways and 62 climate models used in BTV model projection in future climate conditions. (CSV)

S2 File Correlation matrix showing patterns of relationships among environmental variables used in model calibration. (CSV)

S3 File Summary of BTV vector occurrences available for testing niche similarity between BTV and vector niches and based on accessible area (M). (PDF)

S4 File Range of BTV expansion based on presence-absence matrix of each ecological niche model for corresponding climate model. (CSV)

S5 File Results of background similarity tests assessing niche similarity between bluetongue virus and six vector species. The null hypothesis of niche similarity was rejected if the observed D or I values for the BTV and vector species in question fell below the 5^th^ percentile in the random-replicate distribution (i.e. 5% in table).
(PDF)

S6 File Uncertainty estimates associated with BTV mapping process in different climate models within each representative concentration pathway. (PDF)
