# Supplementary figures and images for: Climate Change Influences on the Global Potential Distribution of Bluetongue Virus
Source: PLoS One. 2016 Mar 9;11(3):e0150489. doi: 10.1371/journal.pone.0150489 (PMC4784974; doi:10.1371/journal.pone.0150489)

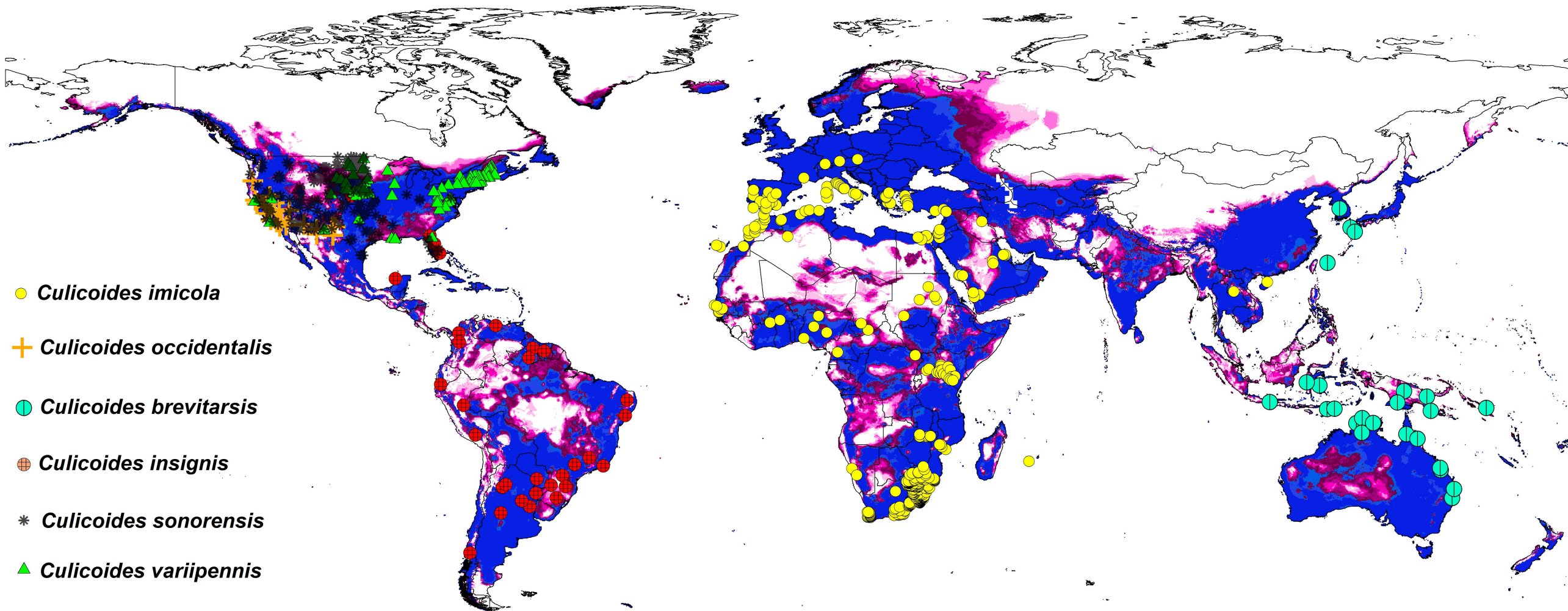

Supplement: S3 File — (PDF) [file pone.0150489.s003.pdf]

RCP 2.6

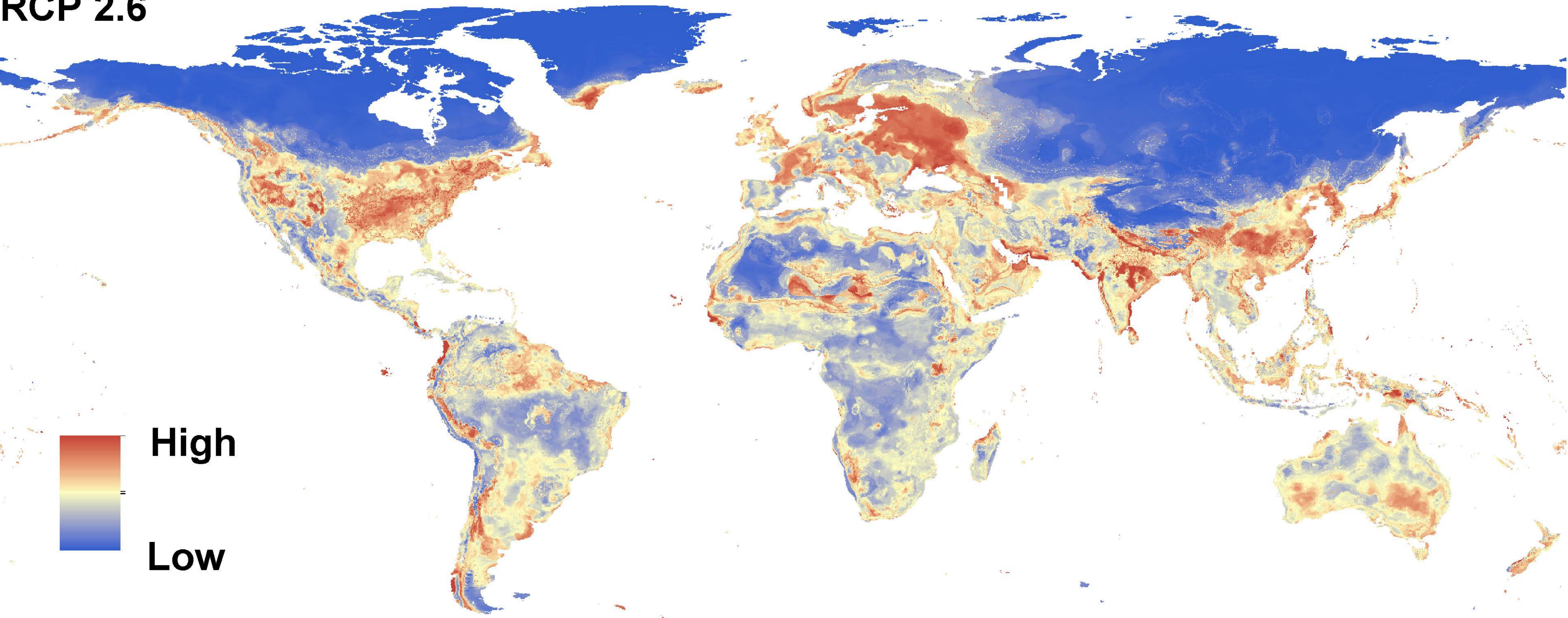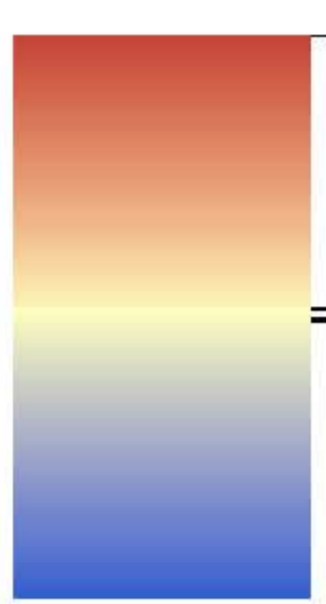

High

Low

RCP 4.5

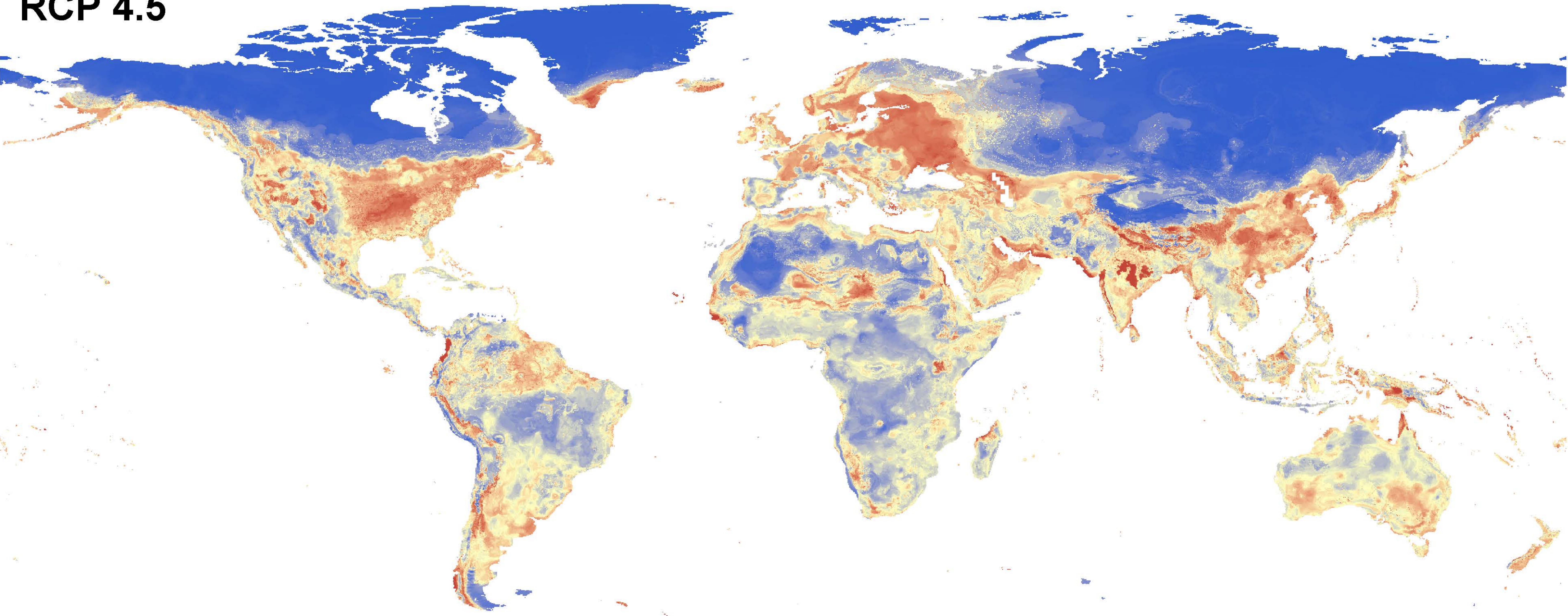

RCP 6.0

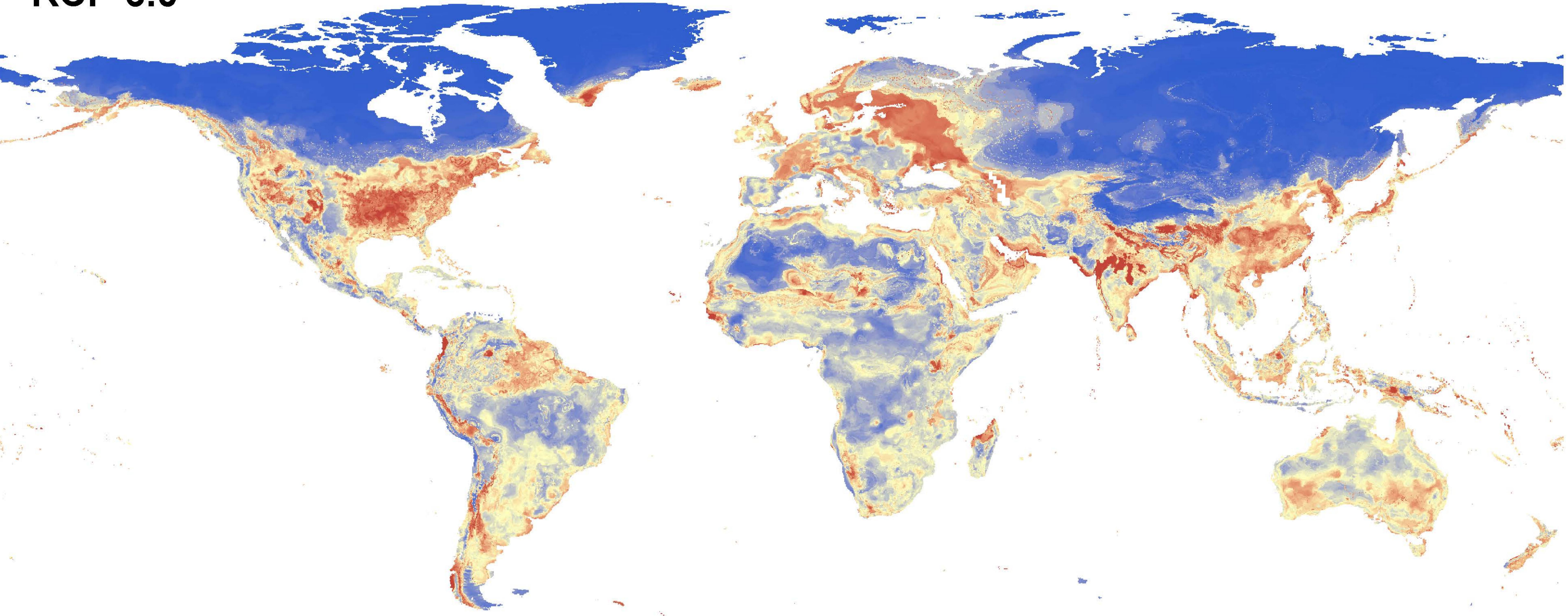

RCP 8.5

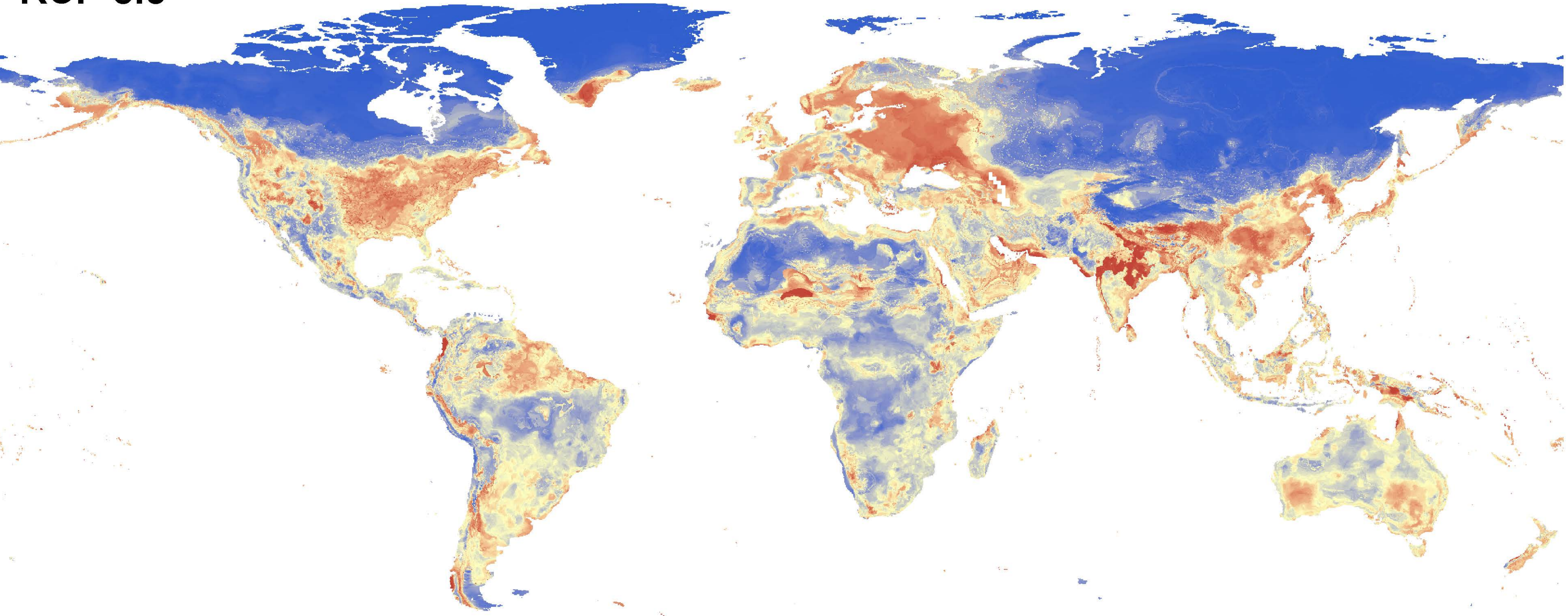

Supplement: S6 File — (PDF) [file pone.0150489.s006.pdf]
