# Supplementary material for: Climate Change Influences on the Global Potential Distribution of Bluetongue Virus
Source: PLoS One. 2016 Mar 9;11(3):e0150489. doi: 10.1371/journal.pone.0150489 (PMC4784974; doi:10.1371/journal.pone.0150489)
Supplement: S5 File — The null hypothesis of niche similarity was rejected if the observed D or I values for the BTV and vector species in question fell below the 5th percentile in the random-replicate distribution (i.e. 5% in table). (PDF) [file pone.0150489.s005.pdf]

S5 File: Results of background similarity tests assessing niche similarity between bluetongue virus and six vector species. The null hypothesis of niche similarity was rejected if the observed *D* or *I* values for the BTV and vector species in question fell the 5th percentile in the random-replicate distribution (i.e. 5% in table).

	Schoener's *D*				Hellinger's *I*			
Species	Observed	5%	95%	*P* value	Observed	5%	95%	*P* value
Culicoides imicola	0.585	0.466	0.863	$P > 0.05$	0.847	0.677	0.926	$P > 0.05$
Culicoides insignis	0.615	0.382	0.683	$P > 0.05$	0.873	0.514	0.944	$P > 0.05$
Culicoides variipennis	0.618	0.419	0.804	$P > 0.05$	0.872	0.615	0.966	$P > 0.05$
Culicoides sonorensis	0.696	0.429	0.717	$P > 0.05$	0.923	0.598	0.958	$P > 0.05$
Culicoides brevitarsis	0.708	0.322	0.942	$P > 0.05$	0.927	0.551	0.956	$P > 0.05$
Culicoides occidentalis	0.505	0.233	0.739	$P > 0.05$	0.805	0.366	0.821	$P > 0.05$
